# High-throughput single-fly LC–MS/MS for quantitative profiling of biogenic amines in *Drosophila*

**DOI:** 10.1371/journal.pone.0341188

**Published:** 2026-01-23

**Authors:** Aki Hori, Takahiro Nitta, Kei-ichiro Inamori, Hirotaka Kanoh, Takayuki Kuraishi

**Affiliations:** 1 Faculty of Pharmacy, Institute of Medical, Pharmaceutical and Health Sciences, Kanazawa University, Kanazawa, Ishikawa, Japan; 2 Division of Glycopathology, Faculty of Pharmaceutical Sciences, Tohoku Medical and Pharmaceutical University, Sendai, Miyagi, Japan; 3 Institute of Molecular Biomembrane and Glycobiology, Tohoku Medical and Pharmaceutical University, Sendai, Miyagi, Japan; 4 Institute for Advanced Biosciences, Keio University, Tsuruoka, Yamagata, Japan; 5 Institute for Frontier Science Initiative, Kanazawa University, Kanazawa, Ishikawa, Japan; 6 Life Science Center for Survival Dynamics, Tsukuba Advanced Research Alliance (TARA), University of Tsukuba, Tsukuba, Japan; 7 JST-FOREST, Japan Science and Technology Agency, Tokyo, Japan; Waseda University: Waseda Daigaku, JAPAN

## Abstract

We present an LC–MS/MS method that enables reproducible, quantitative detection of multiple biogenic amines—including octopamine, dopamine, serotonin, and their precursors—from single *Drosophila* individuals. The workflow involves only a minimal extraction in methanol prior to direct injection, eliminating the need for derivatization or laborious purification steps. Its simplicity ensures high reproducibility and broad applicability across experimental settings. Pilot applications revealed distinct neurochemical responses across biologically relevant conditions: a circadian rise in octopamine at dusk, acute increases in octopamine, dopamine, and serotonin under cold stress, and elevations of octopamine, serotonin, and tryptophan during starvation. These findings highlight the utility of single-head LC–MS/MS for dissecting neuromodulator dynamics at the level of individual organisms.

## Introduction

Biogenic amines (e.g., octopamine, dopamine, and serotonin) function as neurotransmitters or neuromodulators across diverse animal taxa including insects and vertebrates, controlling key physiological and behavioral processes including locomotion, learning and memory, stress responses, and reproduction [1–5]. In insects, octopamine in particular serves as a functional analog of norepinephrine and has been studied extensively in the context of neuromodulatory signaling [1].

The accurate quantification of monoamines in small model organisms like fruit fly, *Drosophila melanogaster*, remains technically demanding due to the small sample size and low endogenous concentrations. Traditional methods—including high-performance liquid chromatography (HPLC) with electrochemical or fluorescence detection and gas chromatography–mass spectrometry (GC–MS)—often require labor-intensive workflows and pooling of multiple individuals [6–9]. Moreover, interference from endogenous charged compounds and the need for high oxidation potentials can limit the sensitivity of electrochemical detection [10]. Liquid chromatography–tandem mass spectrometry (LC–MS/MS) has emerged as a powerful alternative, offering high specificity and sensitivity without derivatization [10,11]. Several recent studies have applied LC–MS/MS to the analysis of octopamine and related monoamines in homogenized insect tissue [12,13]. Among these, the method described by Davla et al. [12] demonstrated simultaneous detection of multiple monoamines from single *Drosophila* heads using an LC–MS/MS platform. While this study was a significant step forward in single-animal analysis, octopamine quantification remained semi-quantitative. Pantalia et al. [14] applied LC–MS/MS to profile neurotransmitters in the heads of ebony mutants, showing reduced levels of dopamine, serotonin, histamine, and octopamine. Similar LC–MS/MS-based workflows have also been employed in other model organisms, including zebrafish [15] and mouse brain [16], further demonstrating the versatility of this analytical platform across species.

In this study, we present an optimized LC–MS/MS method that enables direct, quantitative detection of octopamine along with dopamine, serotonin, L-DOPA, tyramine, tyrosine, and tryptophan from a single *Drosophila*. While LC–MS/MS–based methods for monoamine detection have been reported previously, the primary contribution of the present study lies in the combination of minimal sample preparation, high reproducibility, and quantitative detection of octopamine at the single-fly level. By utilizing a minimal preparation workflow—methanol-based extraction followed by centrifugation and direct injection—we eliminate the need for derivatization or multi-step cleanup. This streamlined protocol enhances sample recovery and reproducibility while achieving sufficient sensitivity for accurate quantification, even for low-abundance analytes like octopamine. This approach expands the experimental feasibility of monoamine profiling in small insects, facilitating behavioral and physiological investigations at the single-animal level.

## Materials and methods

### Drosophila maintenance

Oregon R flies were used as the wild-type strain. Flies were maintained on standard glucose-cornmeal-yeast medium (standard medium) [17]. For preparing 10 L of the standard medium, 800 g baker’s yeast (Asahi Group Foods, Tokyo, Japan) and 400 g corn flour (NIPPN, Tokyo, Japan) were mixed with 4 L deionized water. Separately, 55 g agar (FUJIFILM Wako, Osaka, Japan) was mixed with 6 L deionized water. When the agar solution was heated to 40°C, 1,000 g glucose (Kanto Chemical, Tokyo, Japan) was added and mixed well. This mixture was continuingly heated to 75°C and the yeast and corn flour mixture was added to it. When the solution reached 80°C, heating was stopped and 38 mL propionic acid (FUJIFILM Wako) and 48 mL of 15% butyl 4-hydroxybenzoate (Tokyo Chemical Industry, Tokyo, Japan) were added and mixed. Next, the medium was dispensed into polypropylene vials (Genesee Scientific, El Cajon, CA, USA) and used for experimentation. Flies were maintained at 25°C under a 12:12 h light–dark (LD) cycle (lights on at 07:30 and lights off at 19:30; local time) unless otherwise stated.

### Chemicals

The reference standards used in this study were as follows: octopamine hydrochloride (TCI, O0413), 3-(3,4-dihydroxyphenyl)-L-alanine (L-DOPA; TCI, D0600), dopamine hydrochloride (Nacalai Tesque, 14212–84), tyramine hydrochloride (TCI, A0303), 5-hydroxytryptamine (5-HT; FUJIFILM Wako, 324–42331), L-tyrosine (Sigma-Aldrich, T3754-50G), and L-tryptophan (Sigma-Aldrich, T0254-25G). The buffer reagent 2-morpholinoethanesulfonic acid (MES; TCI, M0606) was also used. LC/MS-grade solvents including methanol (FUJIFILM Wako, 134–14523), acetonitrile (FUJIFILM Wako, 018–19853), and 2-propanol (FUJIFILM Wako, 164–25533) were purchased from FUJIFILM Wako. Primary stock solutions of unlabeled chemicals and an internal standard were prepared as listed: MES; 100 mM in water, OA 100 mg/ml, DA 100 mg/ml, TyA 23 mg/ml, L-DOPA 1 mg/ml, 5-HT 1 mg/ml, Tyr 10 mg/ml (1M HCl), Trp 10 mg/ml. The stock solutions were stored in the dark at −20°C. Individual sub-stocks were prepared at 1 mg/ml in water. Further, dilutions of these sub-stocks (monoamines/amino acids 100, 10 μg/ml, MES 10 µM, in MeOH) were prepared prior to each batch of sample analyses and used to tune the mass spectrometer and were prepared just prior to each standard curve/assay. Met sulfone and 2-morpholinoethanesulfonic acid (MES) were used for an internal standard.

### Lysate preparation from single fly or head

Single *Drosophila* whole body or head were prepared under CO_2_ anesthetization by cutting a head with forceps and put into 50 µL MeOH on dry-ice/ethanol. A whole body or head were then homogenized with a bath-type sonication, and immediately frozen by liquid nitrogen and stored at −80°C. Just before LC-MS/MS analysis, the homogenate was supplemented with internal standards (MES) to a final concentration of 1 μM, sonicated again and centrifuged at 15000 rpm for 10 min at 4°C, the supernatant was collected and used for the analysis.

### LC-MS/MS analysis

Monoamines were measured by ultraperformance liquid chromatography–tandem mass spectrometry (LCMS-8060, Shimadzu Corporation). The interface voltage was 4000 V, the drying gas 10 L/min, heating gas 10 L/min, Nebulizing gas flow 2.0 L/min, the interface temperature 300°C, Desolvation temperature 526°C, Desolvation line (DL) temperature 250°C, heat block temperature 400°C, the CID gas was 230 kPa, and the dwell time and the MRM transitions were listed in the Table 1 for each compound. The Q1 resolution full width half maximum (FWHM) was “High” and the Q3 Resolution (FWHM) was “Unit”. The instrument ran in a positive ion detection mode at various collision energies (CE in V) for the MRM transitions outlined in Table 1.

**Table 1:** Optimized Mass transitions and Corresponding Collision Energies and Retention Times.

Analyte	Mass (M + H+)	MRM transition (Q1/Q3)	Collision Energy (volts)	Retention Time (min)	Dwell Time (msec)
Octpamine	154.1	136.1/91.0	−20	1.120	45
L-DOPA	198.2	198.2/152.0	−20	1.250	45
Dopamine	154.1	154.1/137.0	−15	1.135	45
Tyramine	138.1	138.1/121.0	−15	1.800	45
5-HT	177.2	177.2/160.0	−20	2.450	45
Tyrosine	182.2	182.2/136.0	−15	1.800	10
Tryptophan	205.2	205.2/188.0	−10	4.450	10
MES	196.1	196.1/100.0	−20	1.050	45

Chromatography of monoamines on a C8 analytical column (00F-4497-AN Kinetex C8 column (2.6 µm, 100 Å, 150 × 2.1 mm; Phenomenex, Torrance, CA, USA)) involved adaptation of a method using gradient elution of a binary solvent system that incorporated (A) 0.3% FA (aqueous) and (B) 50% ACN + 50% isopropanol as follows: 0–2.0 min, 0% B; 2.0–5.0 min, 100% B; 5.0–14.9 min, 100% B; 14.9 − 15.0 min, 0% B; 15.0 − 20.0 min 0%. The solvent flow rate was 300 µL/min. A 0.5 µL or 1.0 µL injection volume for single head or a whole body, respectively, was used. The LabSolutions software (Shimadzu Corporation) C8 method file is available in Zenodo as Supplementary_File_1.lcm.

Calibration curves were prepared by spiking standard monoamines and metabolites into 0.5 µL of methanol at final concentrations of 0, 1.0, 10, 100, and 1000 ng/mL. For tyrosine and tryptophan, additional concentrations of 2500 and 5000 ng/mL were also used. Three replicates at each level of standard were injected and the CV% among replicate injections was calculated for each standard. For each analyte, calibration curves were generated (S2 Fig) and the results were calculated. Intra-assay variability (CV%) were calculated from a whole fly or single head extract.

### Circadian rhythm, cold and starvation stresses

For circadian sampling, flies were kept under the 25°C, 12:12 h LD schedule described above and collected at four time points: 07:00, 13:00, 19:00, and 01:00 (Japan Standard Time). For cold exposure, flies were transferred to a 12°C incubator for either 2 h (from 07:00–09:00) or 24 h (from 17:00–17:00 on the following day) and were immediately collected at the end of each exposure. For starvation, flies were subjected to complete food and water deprivation for 12 h (from 09:00–21:00) and collected at 21:00. The number of biological replicates for each condition is indicated in the figure legends.

### Statistical analysis

Statistical analyses were performed in GraphPad Prism 10 (GraphPad Software, La Jolla, CA, USA). The sample numbers were determined empirically. All data points were biological, not technical, replicates. No data were excluded. Bar graphs show mean ± standard error of the mean with all data points of biological replicates, and some graphs show median or mean values as indicated in figure legends. For multiple group comparisons, the Kruskal–Wallis test was applied, followed by Dunn’s multiple comparisons test to identify significant pairwise differences. For two-group comparisons, the Mann–Whitney test was employed. *P < 0.05; **P < 0.01; ***P < 0.001; ****P < 0.0001; ns, not significant.

## Results and discussion

### Simultaneous quantification of biogenic amines and related compounds

We first aimed to develop a method capable of simultaneously detecting key monoamine neurotransmitters and related compounds in a single fly quantitatively, given that individual-level analysis is often desirable for understanding the variability in neuronal signaling. From the range of biologically important monoamines, we selected octopamine (OA), dopamine (DA), and serotonin (5-HT) as our primary targets due to their well-established roles in invertebrate behavior and physiology. In addition, we included several precursors or metabolites—tyrosine (Tyr), tyramine (TA), L-DOPA, and tryptophan (Trp)—to gain a more comprehensive overview of biogenic amines in individual samples.

Because high-throughput measurements require a consistent yet time-efficient workflow, we evaluated various sample preparation strategies to simplify tissue extraction. In order to minimize handling steps and reduce variability, a simple protocol of homogenization of each fly in methanol followed by no additional purification steps was tested. We reasoned that, with this simplified extraction, a large number of samples could be processed consecutively. Another crucial aim was to achieve robust, stable measurements over multiple injections. We sought chromatographic conditions that would provide adequate separation of the highly polar target compounds while allowing a brief runtime and sufficient reconditioning intervals. We therefore opted for a C8 reversed-phase column, which aids in retaining these polar monoamines yet elutes them early enough to incorporate ample wash time at the end of each run. This design may ensure minimal carryover and consistent performance across large sample batches.

Guided by these requirements, we tested extracts containing all target molecules directly in our LC–MS/MS system, comparing theoretical fragment ions to experimental spectra. Following the confirmation of the ability to detect each compound with authentic standards, we optimized the mass spectrometric parameters, including precursor and product ion selections for multiple reaction monitoring (MRM). Our optimization criteria were twofold: obtaining sufficiently high signal intensity and avoiding signal bleed-over into other MRM channels. By systematically refining these settings, we decided the set of MRM transitions, collision energies, and retention times shown in Table 1.

**Fig 1:**
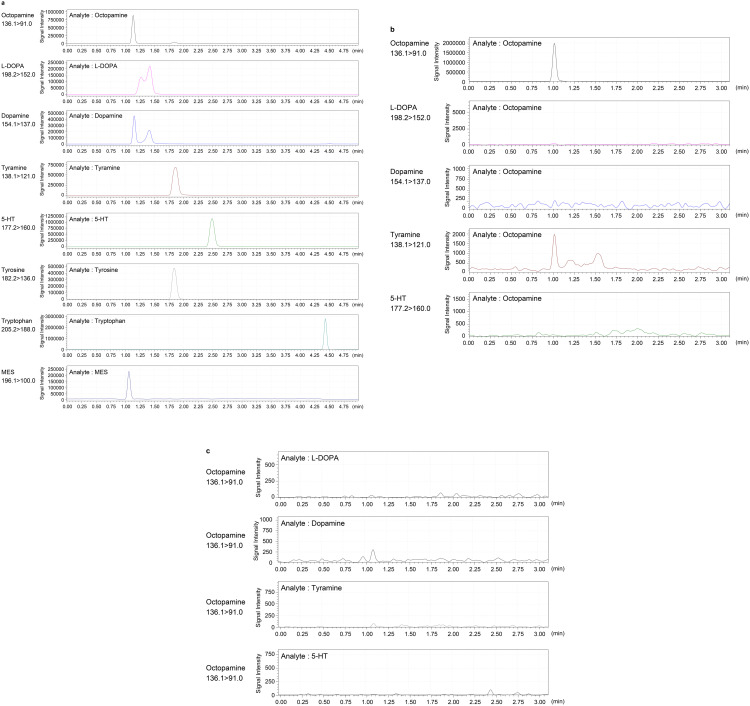
Representative results of mass spectrometric analysis of reference standards. **(a)** MRM chromatograms obtained by LC–MS/MS analysis of reference standards (1 ng/mL, 1 µL injection). Channels: Octopamine, (m/z 136 → 91), L-DOPA (m/z 198.2 → 152), Dopamine (m/z 154.1 → 137), Tyramine (m/z 138.1 → 121), 5-HT (m/z 177.2 → 160), Tyrosine (m/z 182.2 → 136), Tryptophan (m/z 205.2 → 188), and MES, (m/z 196.1 → 100). The x-axis (retention time, min) is displayed for MES and is identical for all chromatograms shown. Using a 1 ng injection standard, we verified that the signal for each compound did not leak into any other channel at a level above 1/1000 of the primary signal (Fig 1b, 1c and S1 Fig). **(b)** Injection of an octopamine standard, monitored across all channels (except for tyrosine and tryptophan) listed in **(a)**. **(c)** Injection of individual reference standards as indicated on the left of the chromatograms, showing no detectable signal in the octopamine (m/z 136 → 91) channel.

Fig 1a presents exemplary MRM chromatograms.

In the case of L-DOPA, the chromatograms displayed a reproducible double-peak profile, which likely compromises quantification accuracy relative to the other monoamines.

We next assessed the method’s sensitivity and linearity. Calibration curves were constructed over relevant concentration ranges (e.g., 1–1000, 10–1000, or 10–5000 ng/mL), all of which exhibited excellent linearity (R^2^ ≥ 0.99) (S2 Fig). The limits of detection (LODs), defined here as the lowest injected amount that produced a clear peak above baseline noise under our MRM conditions, were as follows: 0.5 pg (corresponding to an injection of 0.5 µL of a 1 ng/mL standard solution) for OA, DA, TA, 5-HT, Tyr, and Trp; and 5 pg (corresponding to 0.5 µL of a 10 ng/mL standard solution) for L-DOPA. Moreover, repeated injections (n = 36) of the same sample revealed that measurement variability (coefficient of variation, CV) remained within a few percent for all target analytes (except for dopamine and tyramine, which exceeded 10%) (S3a Fig), suggesting that no significant drift occurred during continuous operation. We also verified that head-only samples yielded results consistent with whole-fly preparations; however, tyramine levels were only reliably detectable in a semi-quantitative manner (data not shown). Repeated measurements of single-head extracts (n = 24) demonstrated stable performance across runs (S3b Fig). These results confirm that our analytical setup is well suited for high-throughput measurements, providing reproducible quantification of monoamines and their precursors or metabolites in single-whole-fly or single-head extracts.

Overall, these results demonstrate that the combination of a minimal methanol-based extraction protocol and C8 reversed-phase chromatography is time-efficient and robust for the routine quantification of multiple monoamines. In particular, we achieved quantitative detection of octopamine, which had previously only been analyzed in a semi-quantitative manner [12], using a simplified extraction protocol suitable for high-throughput analyses. By avoiding extensive cleanup or derivatization steps, we minimized potential sources of loss or variability, and our repeated injections confirmed that the system can handle large numbers of samples sequentially without compromising data quality. Although direct head-to-head comparisons with other analytical methods would be informative, differences in sample requirements and extraction protocols make such comparisons nontrivial; therefore, we rely on analytical validation (linearity, defined LODs, and low CVs) to demonstrate the sensitivity and robustness of the method.

### Biological applications: Circadian rhythm, low-temperature and starvation stresses

The single‑head LC‑MS/MS platform allowed us to quantify neurotransmitters and their precursors—octopamine (OA), dopamine (DA), serotonin (5‑HT), and their biosynthetic precursors tyrosine (Tyr) and tryptophan (Trp)—under three biologically relevant conditions.

#### Circadian rhythm.

**Fig 2:**
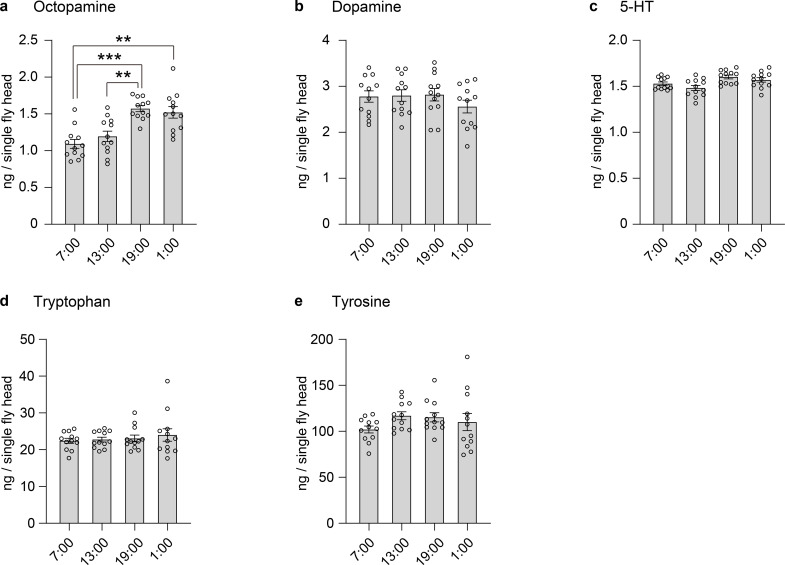
Circadian variation of biogenic amines in Drosophila heads. **(a)−(e)** Concentrations (ng per single fly head) of each compound were measured at four time points (7:00, 13:00, 19:00, and 1:00). Bars indicate mean values± standard error of the mean (s.e.m), and open circles represent individual measurements from 12 fly heads. Data were analyzed using the Kruskal–Wallis test followed by Dunn’s multiple comparisons test.

In our study, among the compounds tested, only OA exhibited clear daily fluctuation, with levels rising in the late light phase (end of photoperiod) (Fig 2a).

This elevation coincided with the empirically observed peak in aggressive behavior with time-of-day specificity in flies. While direct reports of a circadian peak in *D. melanogaster* aggression are limited, a related species (*D. suzukii*) shows time-of-day-dependent aggression, with both males and females displaying high-intensity aggression at a specific photoperiod time [18]. Given OA’s established role as an arousal-promoting neuromodulator, this temporal pattern of OA elevation may represent a clock-regulated anticipatory signal that primes animals for competitive encounters. Consistent with this idea, octopamine in insects is functionally analogous to norepinephrine in mammals, modulating arousal and aggressive readiness [19]. Thus, a dusk-associated OA peak could plausibly prepare male flies for the surge in social interactions (aggression or courtship) that occurs during their active evening period. While the present study focuses on circadian OA dynamics in *D. melanogaster*, extending this single-head LC–MS/MS approach to closely related species such as *D. suzukii* will be an important future application for comparative and evolutionary analyses.

By contrast, neither DA nor 5-HT exhibited significant diurnal changes in our assay (Fig 2b–c), consistent with prior findings that dopamine content in the *Drosophila* brain does not exhibit circadian variation [20]. Similarly, 5-HT levels were stable throughout the light–dark cycle (Fig 2c). While serotonergic inputs to clock neurons have been implicated in photic entrainment and sleep architecture [21], bulk 5-HT content does not seem to follow an overt circadian rhythm. Notably, both Tyr and Trp, as biosynthetic precursors, showed no significant fluctuation across timepoints in our assay (Fig 2d–e). This observation is consistent with recent metabolomic data showing minimal circadian variation in Trp levels in *Drosophila* brain tissue [22]. For Tyr, while modest diurnal differences have been reported [22]—suggesting slightly higher levels during the light phase—such changes were relatively small in magnitude and may reflect differences in sampling scope, such as the use of whole heads versus isolated brains. Overall, the temporal stability of Tyr and Trp in our dataset likely reflects tight metabolic control to ensure continuous availability for neurotransmitter biosynthesis throughout the day–night cycle.

#### Cold stress.

**Fig 3:**
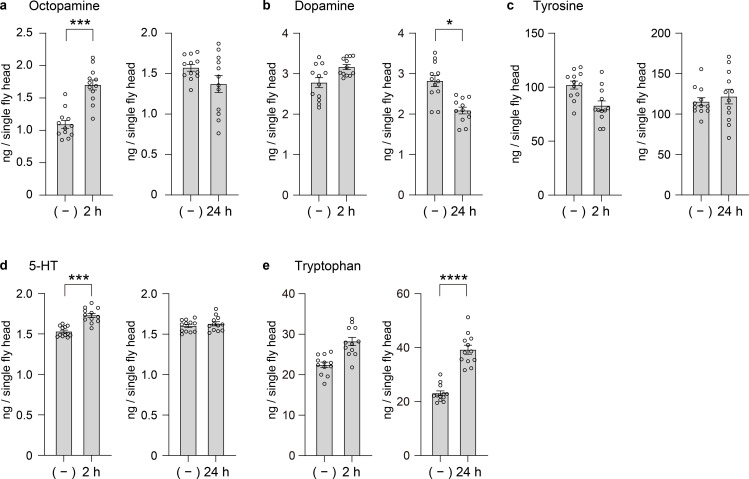
Effects of cold stress on biogenic amine levels in Drosophila heads. **(a)−(e)** Concentrations (ng per single fly head) of each compound were measured under control and cold-stress conditions. Bars indicate mean ± s.e.m, and open circles represent individual measurements (n = 12 heads). Left, (–) circadian 7:00 (same data as in Fig 2) and after 2 h at 12°C starting from 7:00 (sample collected at 9:00); Right, (–) circadian 19:00 (same data as in Fig 2) and after 24 h at 12°C starting from 17:00 (sample collected at 17:00 the next day). Here, (–) denotes circadian control values. The circadian data (Fig 2) are included here as controls for direct comparison with cold-stress conditions. Data were analyzed using the Kruskal–Wallis test followed by Dunn’s multiple comparisons test.

Upon exposure to 12°C for 2 hours, OA, DA, and 5-HT levels all increased significantly in our assay (Fig 3a–c).

The rapid elevation of OA aligns with its established role in the rapid cold hardening (RCH) response observed in various insects. In the migratory locust (*Locusta migratoria*), OA contributes to RCH by helping to stabilize K⁺ homeostasis in the central nervous system, thereby delaying the onset of spreading depolarization and preventing chill-coma [23]. A similar neuroprotective mechanism has been described in *Drosophila*, where OA signaling modulates glial buffering of extracellular K⁺ and water transport, maintaining ionic balance during thermal stress [24].

The cold-induced rise in DA after 2 hours suggests an acute neuromodulatory response, potentially mediated by enhanced synthesis or mobilization of stored DA. However, this elevation is transient: after 24 hours of cold exposure, DA levels decline markedly (Fig 3b). This biphasic pattern may resemble neuroendocrine dynamics observed in cold-stressed insects. In *Alphitobius diaperinus*, Lalouette et al. reported a reduction in Tyr levels following cold exposure [25] as we observed (Fig 3c), which was interpreted as a consequence of increased synthesis of stress-related hormones such as DA. Although DA levels were not directly measured in their study, the decline in Tyr supports the hypothesis of enhanced catecholamine production in the early phase of cold stress. The subsequent decrease in DA observed in our study (Fig 3b) may reflect a later suppression of dopamine biosynthesis, potentially involving reduced tyrosine hydroxylase activity or other regulatory mechanisms.

5-HT also increased acutely under cold exposure (Fig 3d), consistent with prior evidence that 5-HT can support cardiac function and stress resilience under cold shock in *Drosophila* larvae [26]. Cold exposure may trigger systemic release of 5-HT to maintain circulation and neural activity during environmental perturbation. Interestingly, Trp levels also rose significantly following cold exposure (Fig 3e). Since protein synthesis is generally downregulated at low temperatures, the observed increase in Trp may reflect proteolysis coupled with reduced anabolic incorporation, providing a surplus of precursor for enhanced 5-HT or melatonin synthesis [27]. The lack of change in Tyr levels under the same conditions (Fig 3c) further emphasizes the differential regulation of amino acid metabolism in response to acute cold stress.

#### Starvation stress.

**Fig 4:**
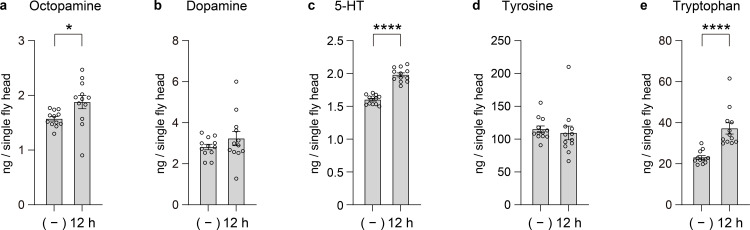
Effects of starvation stress on biogenic amine levels in Drosophila heads. **(a)−(e)** Concentrations (ng per single fly head) of each compound were measured under control and starvation conditions. Bars indicate mean ± s.e.m., and open circles represent individual measurements (n = 12 heads). The x-axis shows two conditions: (–) circadian 19:00 (data identical to Fig 2) and 12 h of starvation (food and water deprivation) starting from 7:00. The circadian data (Fig 2) are included here as a control for direct comparison with starvation stress. Significance was determined using the Mann–Whitney test.

Twelve hours of complete food and water deprivation led to increased levels of OA, 5-HT and Trp, while DA and Tyr showed more nuanced responses (Fig 4a–e).

OA is known to mediate starvation-induced hyperactivity and foraging behavior in *Drosophila*, acting as a central neuromodulator that shifts the behavioral state from energy conservation to resource acquisition [28]. OA-deficient flies fail to show this hyperlocomotor response and instead display abnormal fat metabolism and altered insulin signaling, underscoring its critical role in metabolic adaptation [29].

DA levels remained stable in our measurements (Fig 4b), a finding compatible with models where short-term starvation affects dopamine function through dynamic release and receptor-level modulation rather than changes in overall abundance. For instance, Inagaki et al. reported that six hours of food deprivation in *Drosophila* enhanced dopamine signaling in specific gustatory circuits without increasing dopamine activity in higher brain regions such as the mushroom bodies [30]. In this context, DA may act as a transient synaptic modulator, with behavioral effects governed by dynamic release and receptor sensitivity rather than total tissue concentration [31].

5-HT increased under starvation (Fig 4c), which may reflect the activation of specific serotonergic circuits that regulate macronutrient choice and motivational drive. For instance, some 5-HT neurons are known to promote protein hunger, while others suppress general feeding, indicating complex and context-dependent regulation [32]. The overall rise in 5-HT observed here suggests a broad recruitment of these pathways in response to internal nutrient deficits.

While Tyr levels did not change, Trp again rose under starvation (Fig 4d–e), mirroring its response to cold exposure. The increase likely reflects catabolic liberation from muscle proteins and reduced utilization in anabolic pathways. This pool of free Trp may serve to sustain 5-HT synthesis or feed into kynurenine and NAD⁺ biosynthetic pathways critical for stress resilience [33,34].

Taken together, these results highlight the distinct regulatory profiles of monoaminergic compounds in response to circadian, thermal, and nutritional cues. Among them, OA emerges as a centrally regulated neuromodulator whose levels fluctuate dynamically across conditions. Its circadian peak at dusk coincides with increased social activity, while its consistent elevation under cold and starvation stresses supports a role in promoting arousal and adaptive behavioral responses. In contrast, DA levels remain largely stable, suggesting that its behavioral functions may be mediated through rapid synaptic mechanisms rather than broad fluctuations in tissue concentration. 5-HT exhibits marked sensitivity to environmental stressors, increasing under both cold and starvation, potentially reflecting its involvement in metabolic adaptation and motivational drive. The biosynthetic precursors Tyr and Trp display tight homeostatic control under baseline conditions, although Trp is selectively elevated under stress, suggesting its role as a mobilizable pool supporting neuromodulator synthesis.

Together, the circadian, cold-stress, and starvation paradigms illustrate that the single-head LC–MS/MS platform can robustly detect condition-specific changes in biogenic amines across diverse physiological and behavioral contexts.

## Conclusions

The goal of this study was to establish a practical and reproducible LC–MS/MS workflow that enables quantitative monoamine profiling from single flies under diverse experimental conditions. Compared with conventional techniques such as HPLC with electrochemical detection, our single-fly LC–MS/MS platform significantly streamlines sample preparation by eliminating extensive cleanup steps. The use of reversed-phase C8 chromatography and MRM detection achieves high sensitivity and short run times, enabling large-scale analyses of multiple monoamines. Although some LC–MS/MS methods may match or exceed our detection limits, they often require more laborious protocols that can mask subtle biological variations. A key limitation of our approach is that it measures entire heads or whole bodies rather than isolated brains, thus capturing contributions from peripheral tissues (e.g., the fat body or cuticle). Nonetheless, for high-throughput screening and comparative studies, this method offers a practical balance between simplicity and analytical rigor. Application of this single-head LC–MS/MS workflow to monoamine-pathway mutants will be an important future direction to further explore genetically defined alterations in neurotransmitter pools.

Three pilot applications of our method illustrate how our single-head LC–MS/MS platform can reveal distinct profiles of neurotransmitters and their precursors under biologically relevant conditions. Our findings demonstrate the power of our single-head, multi-analyte LC-MS/MS platform to resolve condition-specific patterns in neuromodulator regulation. Future studies leveraging this approach may elucidate causal links between monoamine dynamics and behavioral output at the level of individual organisms.

## Supporting information

S1 Fig Specificity of individual MRM channels. (a) L-DOPA channel (m/z 198.2 → 152), (b) Dopamine channel (m/z 154.1 → 137), (c) Tyramine channel (m/z 138.1 → 121), and (d) 5-HT channel (m/z 177.2 → 160) were monitored following injection of various reference standards as indicated on the left of the chromatograms. No significant signal leakage was observed into these channels.
(PDF)

S2 Fig Calibration curves for reference standards. (a)−(g) Dose–response curves for each compound were obtained by plotting MES-normalized signal intensity against concentration. Linear regression equations and coefficients of determination (R²) are shown within each panel. The x-axis (ng/mL) is displayed on a logarithmic scale to illustrate the wide dynamic range of quantification.
(PDF)

S3 Fig Reproducibility of repeated measurements of whole-fly extracts. (a)−(g) Signal intensities of each compound were measured consecutively 36 times from a single *Drosophila* extract. Plots show MES-normalized signal intensity (y-axis) against injection number (x-axis), demonstrating stable performance across repeated runs. (h) Summary statistics of all replicates, including mean, standard deviation (SD), and coefficient of variation (CV%). (i)−(n) Signal intensities of each compound were measured consecutively 24 times from a single-head extract. (o) Summary statistics of all replicates, including mean, SD, and CV(%).
(PDF)
