# Supplementary figures and images for: High-throughput single-fly LC–MS/MS for quantitative profiling of biogenic amines in Drosophila
Source: PLoS One. 2026 Jan 23;21(1):e0341188. doi: 10.1371/journal.pone.0341188 (PMC12829853; doi:10.1371/journal.pone.0341188)

Supplementary Fig 1

a

L-DOPA  
198.2>152.0

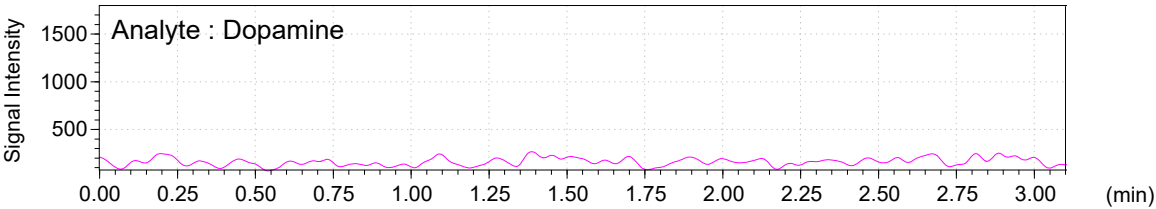

L-DOPA  
198.2>152.0

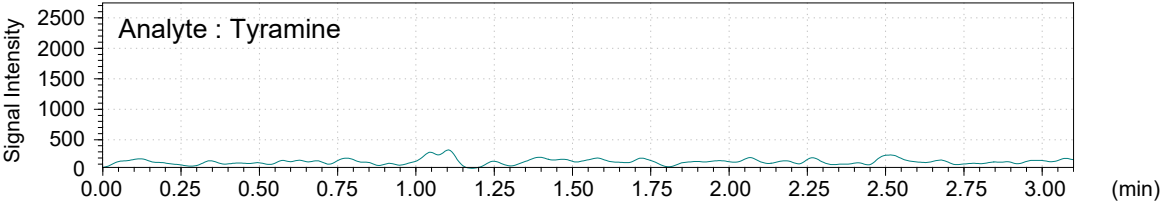

L-DOPA  
198.2>152.0

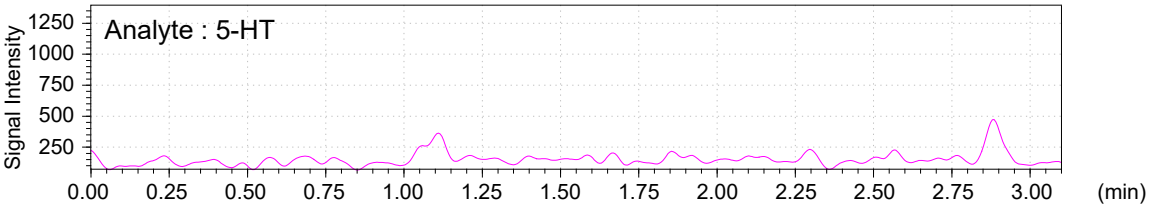

b

Dopamine  
154.1>137.0

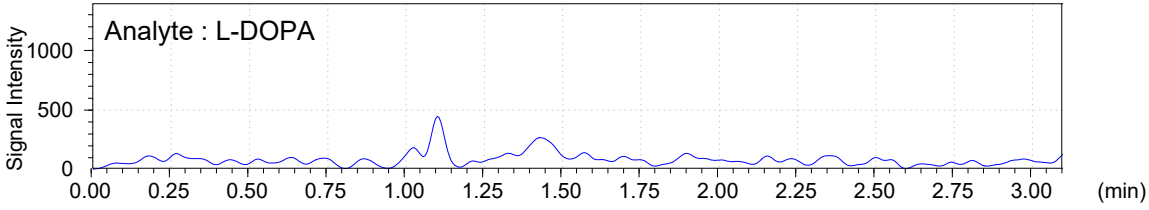

Dopamine  
154.1>137.0

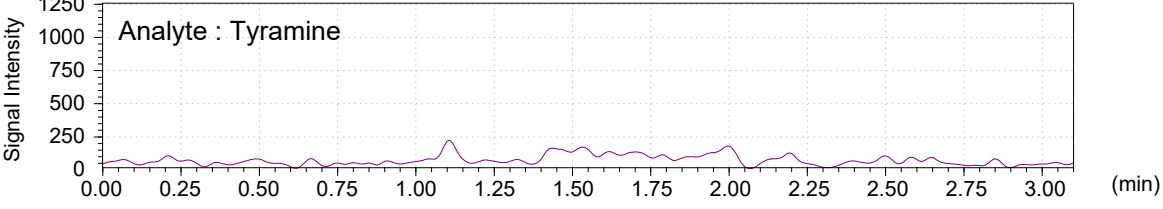

Dopamine  
154.1>137.0

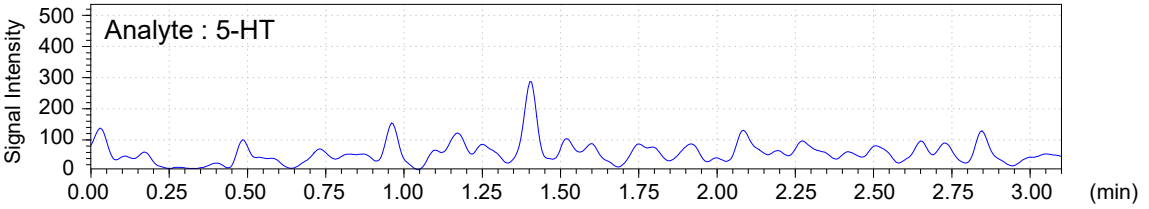

Supplementary Fig 1

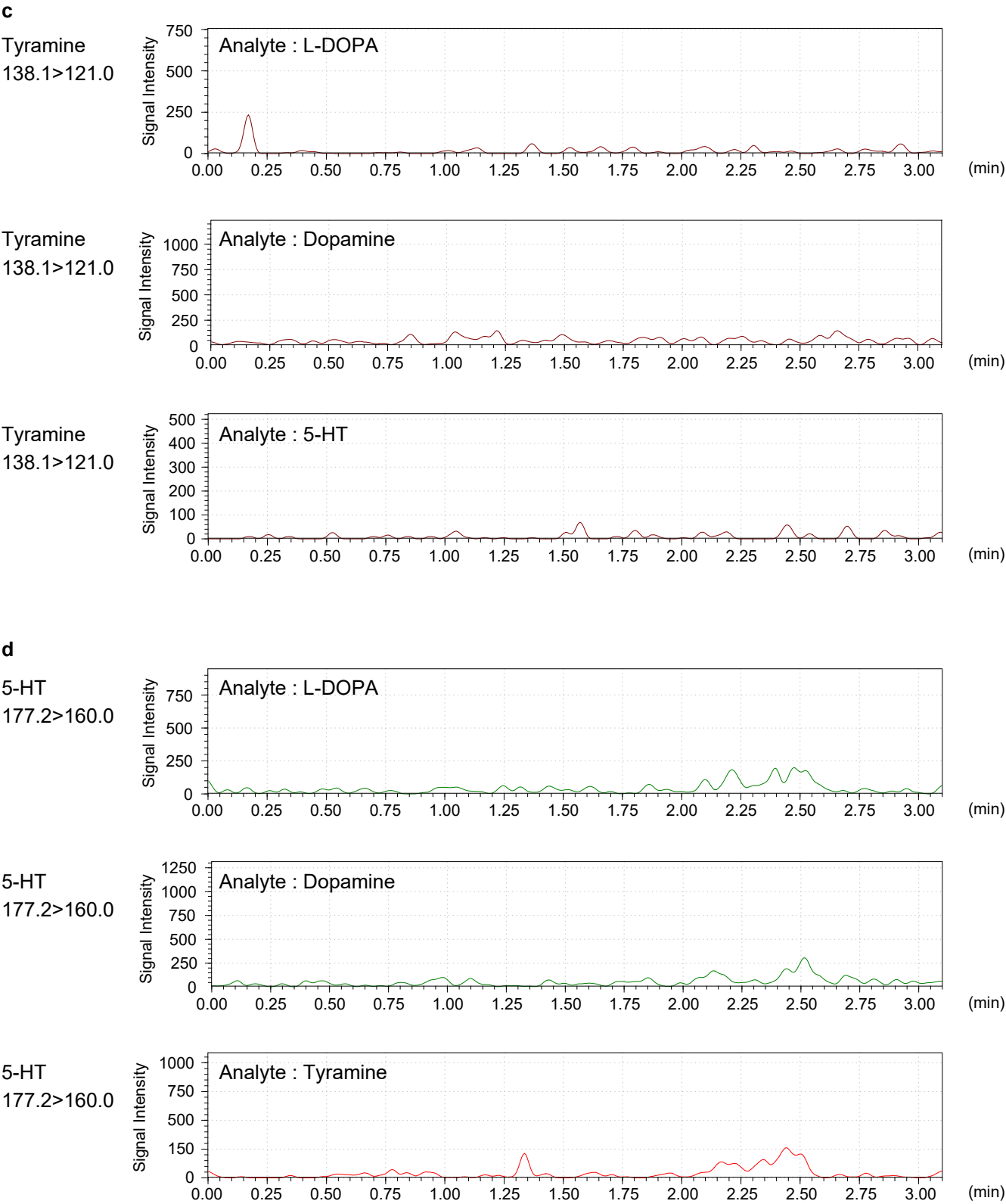

Supplement: S1 Fig — (a) L-DOPA channel (m/z 198.2 → 152), (b) Dopamine channel (m/z 154.1 → 137), (c) Tyramine channel (m/z 138.1 → 121), and (d) 5-HT channel (m/z 177.2 → 160) were monitored following injection of various reference standards as indicated on the left of the chromatograms. No significant signal leakage was observed into these channels. (PDF) [file pone.0341188.s001.pdf]

Supplementary Fig 2

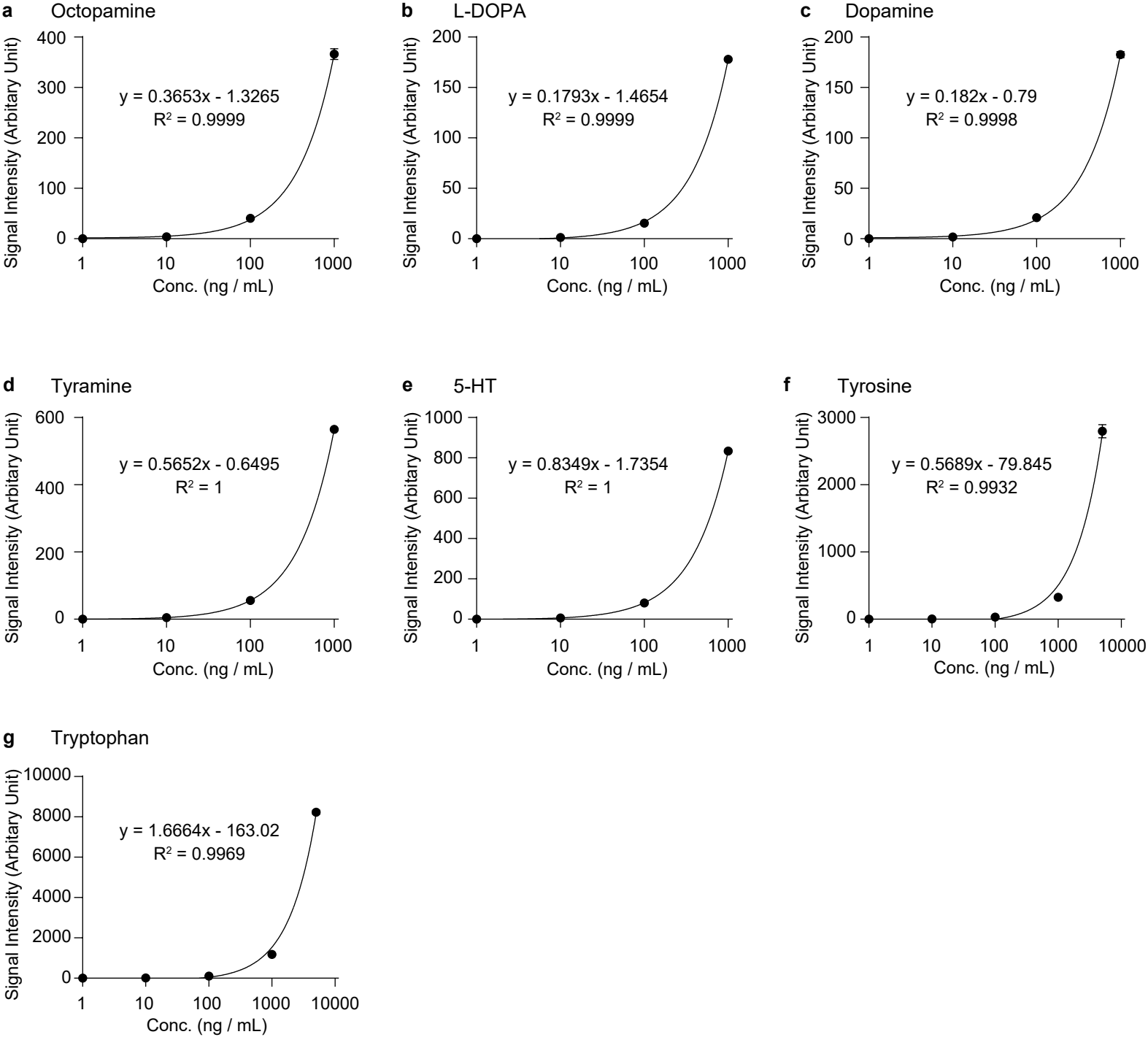

Supplement: S2 Fig — (a)−(g) Dose–response curves for each compound were obtained by plotting MES-normalized signal intensity against concentration. Linear regression equations and coefficients of determination (R²) are shown within each panel. The x-axis (ng/mL) is displayed on a logarithmic scale to illustrate the wide dynamic range of quantification. (PDF) [file pone.0341188.s002.pdf]
