# Supplementary material for: High-throughput single-fly LC–MS/MS for quantitative profiling of biogenic amines in Drosophila
Source: PLoS One. 2026 Jan 23;21(1):e0341188. doi: 10.1371/journal.pone.0341188 (PMC12829853; doi:10.1371/journal.pone.0341188)
Supplement: S3 Fig — (a)−(g) Signal intensities of each compound were measured consecutively 36 times from a single Drosophila extract. Plots show MES-normalized signal intensity (y-axis) against injection number (x-axis), demonstrating stable performance across repeated runs. (h) Summary statistics of all replicates, including mean, standard deviation (SD), and coefficient of variation (CV%). (i)−(n) Signal intensities of each compound were measured consecutively 24 times from a single-head extract. (o) Summary statistics of all replicates, including mean, SD, and CV(%). (PDF) [file pone.0341188.s003.pdf]

Supplementary Fig 3

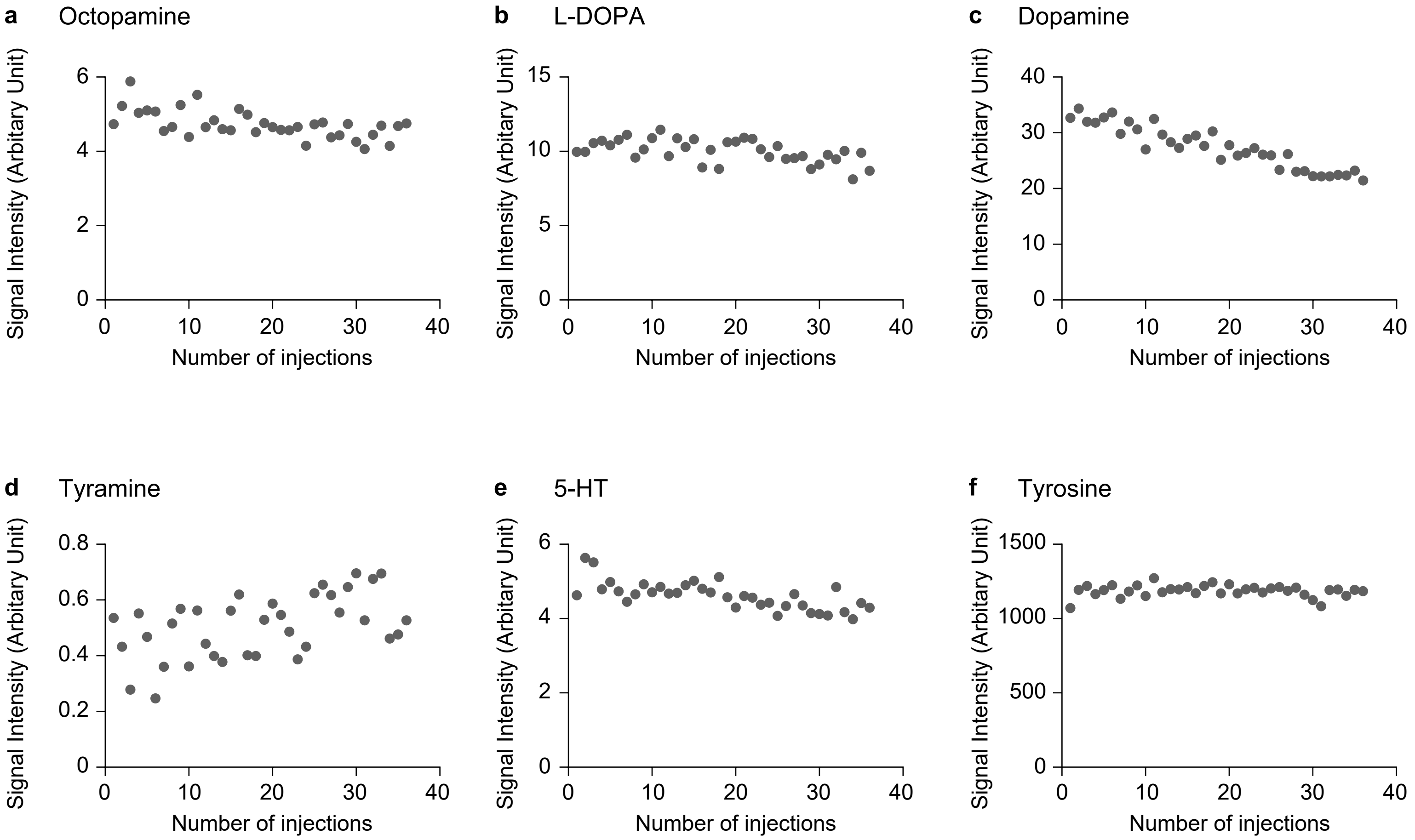

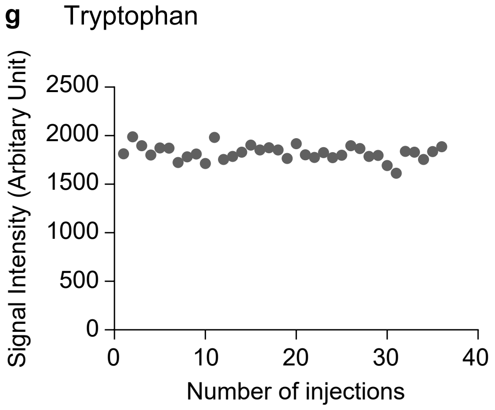

h

	mean	SD	CV (%)
Octopamine	4.73	0.38	8.01
L-DOPA	10.02	0.78	7.75
Dopamine	27.42	3.86	14.07
Tyramine	0.51	0.11	22.47
5-HT	4.61	0.38	8.14
Tyrosine	1185.85	39.91	3.37
Tryptophan	11821.56	75.95	4.17

Supplementary Fig 3

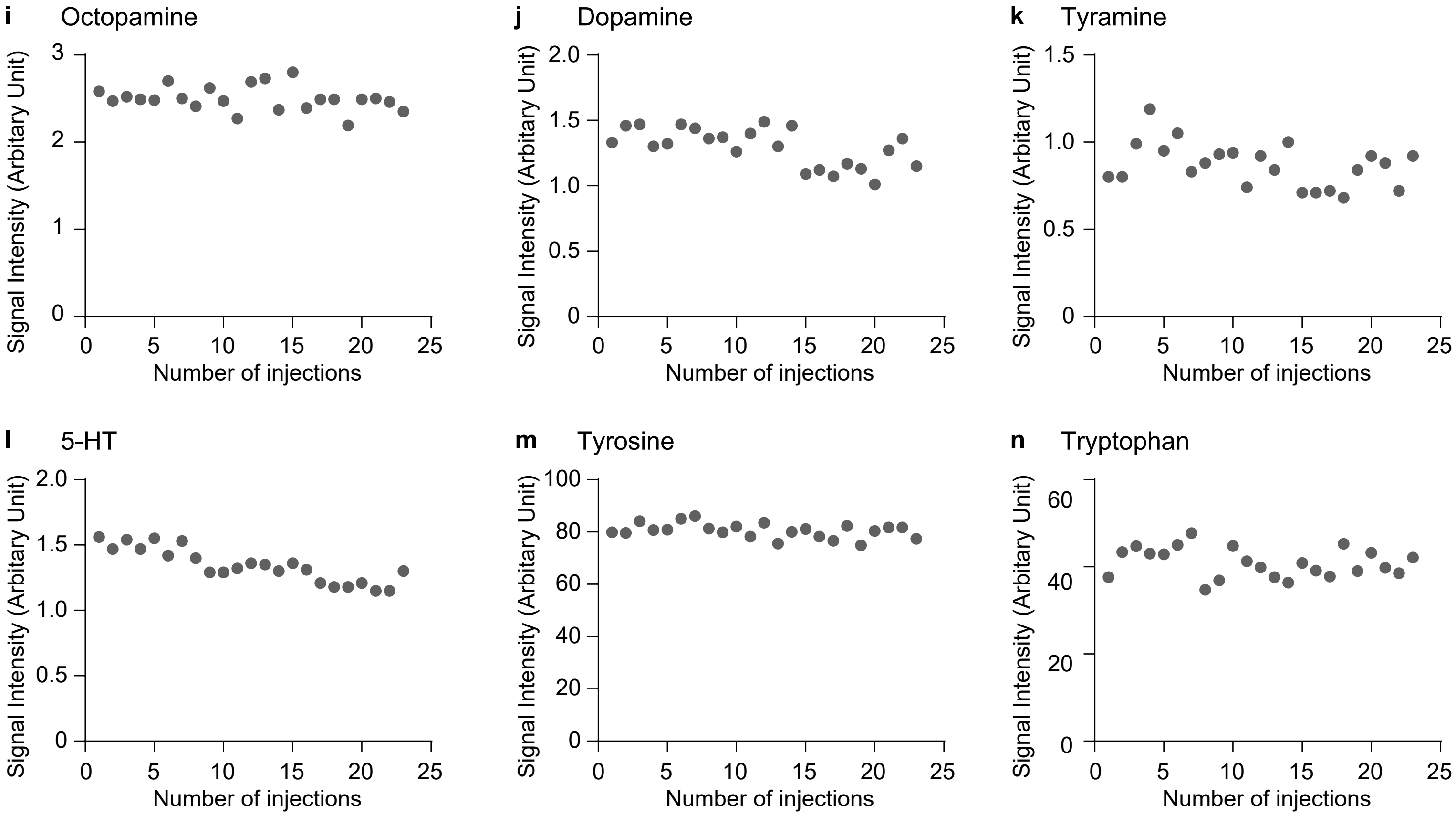

o

	mean	SD	CV (%)
Octopamine	2.49	0.14	5.71
Dopamine	1.26	0.14	11.28
Tyramine	0.86	0.12	14.48
5-HT	1.34	0.13	9.74
Tyrosine	80.46	2.83	3.51
Tryptophan	40.94	3.41	8.34
